# High concentrations of Maraviroc do not alter immunological and metabolic parameters of CD4 T cells

**DOI:** 10.1038/s41598-024-64902-y

**Published:** 2024-06-17

**Authors:** Erick De La Torre Tarazona, Caroline Passaes, Santiago Moreno, Asier Sáez-Cirión, José Alcamí

**Affiliations:** 1 Infectious Diseases Department, Instituto Ramón y Cajal de Investigación Sanitaria (IRYCIS), University Hospital Ramón y Cajal, Madrid, Spain; 2 HIV, Inflammation and Persistence Unit, Institut Pasteur, Université Paris Cité, Paris, France; 3 Viral Reservoirs and Immune Control Unit, Institut Pasteur, Université Paris Cité, Paris, France; 4 AIDS Immunopathogenesis Unit, National Center of Microbiology, Instituto de Salud Carlos III (ISCIII), Madrid, Spain; 5 Centro de Investigación Biomédica en Red de Enfermedades Infecciosas (CIBERINFEC), Instituto de Salud Carlos III (ISCIII), Madrid, Spain; 6 Department of Medicine, Alcalá University, Madrid, Spain

**Keywords:** HIV latency, Latency reversal agents, Maraviroc, Activation markers, Metabolic rates, Immunology, Microbiology

## Abstract

Maraviroc (MVC) is an antiretroviral drug capable of binding to CCR5 receptors and block HIV entry into target cells. Moreover, MVC can activate NF-kB pathway and induce viral transcription in HIV-infected cells, being proposed as a latency reversal agent (LRA) in HIV cure strategies. However, the evaluation of immunological and metabolic parameters induced by MVC concentrations capable of inducing HIV transcription have not been explored in depth. We cultured isolated CD4 T cells in the absence or presence of MVC, and evaluated the frequency of CD4 T cell subpopulations and activation markers levels by flow cytometry, and the oxidative and glycolytic metabolic rates of CD4 T cells using a Seahorse Analyzer. Our results indicate that a high concentration of MVC did not increase the levels of activation markers, as well as glycolytic or oxidative metabolic rates in CD4 T cells. Furthermore, MVC did not induce significant changes in the frequency and activation levels of memory cell subpopulations. Our data support a safety profile of MVC as a promising LRA candidate since it does not induce alterations of the immunological and metabolic parameters that could affect the functionality of these immune cells.

## Introduction

The administration of antiretroviral therapy (ART) effectively suppresses HIV replication, reduces plasma viral load, and delays clinical disease progression^1^. Nevertheless, it is important to note that ART is unable to cure the infection. The need for lifelong ART arises due to viral persistence in HIV latent reservoirs that remain inaccessible to current treatments and undetectable by the immune system^2,3^. This lifelong HIV latent reservoir is quickly established in vivo after infection and primarily consists of memory resting CD4 T cells harboring the viral genome integrated into their DNA^4,5^. Additionally, the latent HIV reservoir may quickly rebound and appear viremia when ART is discontinued^6^.

Current efforts aimed at finding an HIV cure are primarily focused on eradicating or significantly reducing the viral reservoir. In this context, the "shock and kill" strategy has been proposed, which involves the administration of pharmacological compounds known as latency-reversing agents (LRA) to activate viral gene expression and reverse HIV latency^7,8^. Several compounds have demonstrated the capacity to reactivate the HIV latent reservoir, such as histone deacetylase (HDAC) inhibitors (such as vorinostat, romidepsin, Panobinostat), PKC agonists (such as prostratin, deoxyphorbols, bryostatin), Toll-like receptor (TLR) agonists, among others, in both ex vivo^9–12^ and in vivo studies^13–22^. However, significant challenges arise following LRA exposure, as ex vivo assays have shown a wide variation in HIV reactivation responses^10,11^. Additionally, each LRA can modulate the expression of HIV latent reservoirs differently among distinct CD4+ T cell subpopulations^23,24^.

Although various clinical trials have demonstrated the capacity of LRAs to reactivate latent HIV expression in vivo, their administration has often failed to significantly reduce the HIV reservoir size in most of these studies^13,16–20,25^. Moreover, the CD8-mediated cytotoxic response is crucial to eliminate HIV infected cells when LRAs are administered^12^. However, some of these LRAs are employed in cancer treatment and may exhibit toxicity in T cells when were administered at concentrations that activate latent HIV expression^26^. For instance, romidepsin and bryostatin-1 can affect the cytotoxic immune response of primary HIV-1 specific CD8+ T cells, diminishing their capacity to eliminate autologous resting HIV- infected CD4+ T cells^27^. Therefore, it is essential to explore alternative drugs capable of efficiently reactivating HIV latency without toxicity and with a high level of safety to preserve the immunological parameters in patients.

On the other hand, Maraviroc (MVC) is an inhibitor of HIV entry that has received approval for the treatment of patients infected with R5-tropic HIV^28^. MVC inhibits the binding of chemokines CCL3, CCL4, and CCL5 to CCR5, resulting in a potent inhibition of CCR5 transduction signaling without receptor internalization^29^. The drug binds to CCR5 and alters receptor conformation leading to inhibition of gp120 viral envelope binding to CCR5 thereby preventing its entry into the host cell^30–32^. In addition to its antiviral activity, MVC is able to signal through CCR5 and induce viral transcription in HIV-infected cells, with similar or greater potency than other LRAs employed in clinical trials, including the potent PKC agonist bryostatin-1^33–35^. Moreover, MVC activates the NF-κB pathway and subsequently induces the transcription of latent HIV in resting CD4+ T cells from HIV-1-infected individuals on suppressive ART^36,37^. The well-established tolerability and safety profile of MVC make it a strong candidate for consideration as a latency reversal agent (LRA) in the "shock and kill" strategy, either alone or in combination with other LRA. Importantly, at MVC concentrations capable of activating HIV transcription, no alteration of the immune cytotoxic response of CD8 T cells has been observed^34^. However, there is limited data regarding the effect of high concentrations of MVC on CD4 T cells, which are the primary cellular target for HIV infection and crucial modulators of the immune response against viral infections.

The objective of our study was to assess the impact of MVC on immunological and metabolic markers of CD4 T cells, an aspect that has not been previously explored. In this study, we present new data that support the safety of MVC as an LRA candidate for use in strategies aimed at achieving a functional cure for HIV.

## Results

### Lack of significant impact of MVC on activation marker levels in CD4 T cells

**Figure 1:**
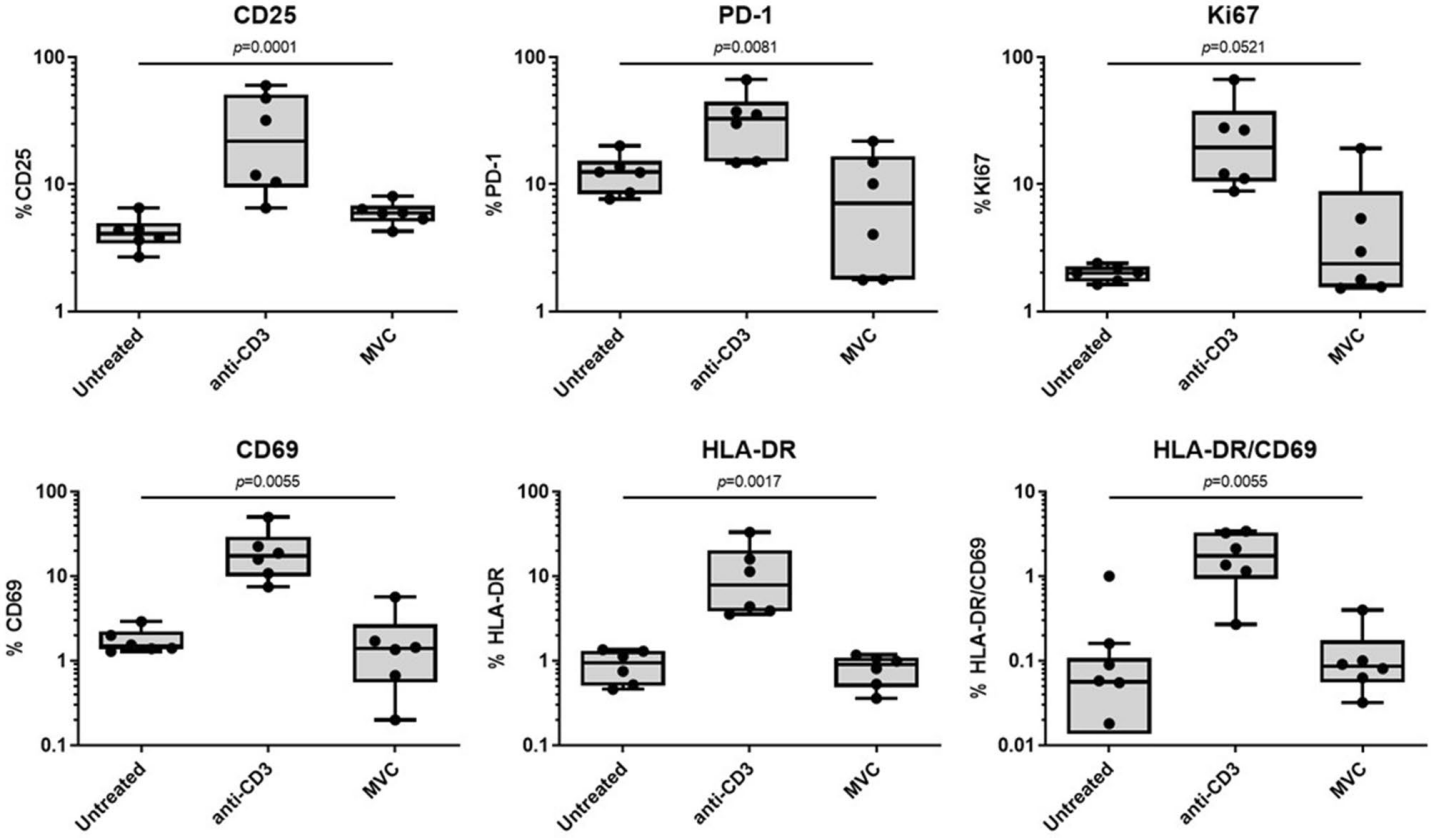
Levels of activation markers in CD4 T cells. Purified CD4 cells were cultured in the absence (untreated) or presence of MVC (5 μM), or anti-CD3 (positive control), during 4 days. T cell activation markers (CD69, HLA-DR, PD-1, Ki67 and CD25) were determined by flow cytometry. Results are shown as percentage of expression of activation markers in CD4 T cells. Values were summarized as median and interquartile range. The statistical analysis among the conditions was performed with the Friedman test. No significant differences between untreated and MVC-treated CD4 T cells were observed.

We examined the influence of MVC on the levels of activation markers in CD4 T cells. Our observations reveal that in vitro MVC did not elicit significant alterations in the levels of activation markers, including CD69, Ki76, PD-1, HLA-DR, and CD25, when compared to untreated cells, unlike the treatment with anti-CD3 antibodies as positive control that elevates the levels of these analyzed activation markers (Fig. 1). Also, analyzing the CD4 T cells expressing HLA-DR and CD69 simultaneously, we observed a similar pattern as observed in individual analysis of these activation markers. Also, Ki67 is considered as a proliferation marker, indicating that MVC did not significantly alter the proliferation of CD4 T cells, as has been previously reported^33^.

### Metabolic parameters of CD4 T cells were not modified in response to MVC stimulation

**Figure 2:**
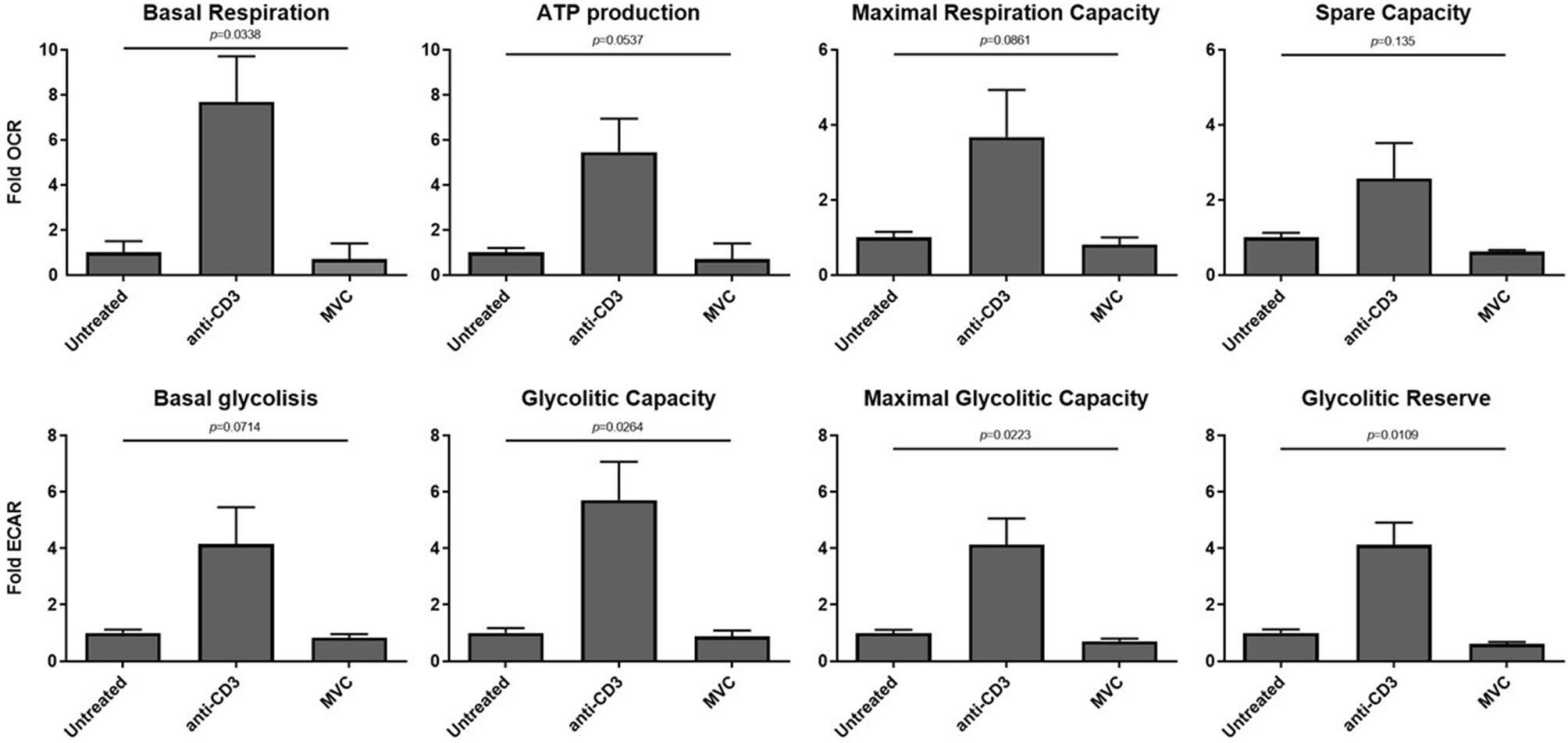
Metabolic parameters of CD4 T cells. Purified CD4 cells were cultured in absence (untreated) or presence of MVC (5 μM), or anti-CD3 (positive control), during 4 days. Metabolic parameters, OCR (oxygen consumption rate) and ECAR (extracellular acidification rate), were measured with a Seahorse XF analyzer. Results are shown as fold change of untreated condition. No differences were found between untreated and MVC conditions in each metabolic parameter. Data are representative of at least three independent experiments. Values were summarized as median and interquartile range. The statistical analysis among the conditions was performed with the ANOVA test. No significant differences between untreated and MVC-treated CD4 T cells were observed.

Finally, we explored the metabolic parameters of CD4 T cells subsequent to MVC stimulation. Our observations demonstrate that MVC did not induce any alterations in the levels of either glycolytic (including basal glycolysis, glycolytic capacity, maximal glycolytic capacity, and glycolytic reserve) or oxidative (including basal respiration, ATP production, maximal respiration capacity, and spare capacity) metabolic rates in comparison to untreated CD4 T cells. This is in sharp contrast with changes produced in anti-CD3 antibodies stimulated cells, which exhibited a considerable increase in several of both glycolytic and oxidative parameters (Fig. 2).

### MVC did not modify the proportion and activation levels of different CD4 memory subsets

**Figure 3:**
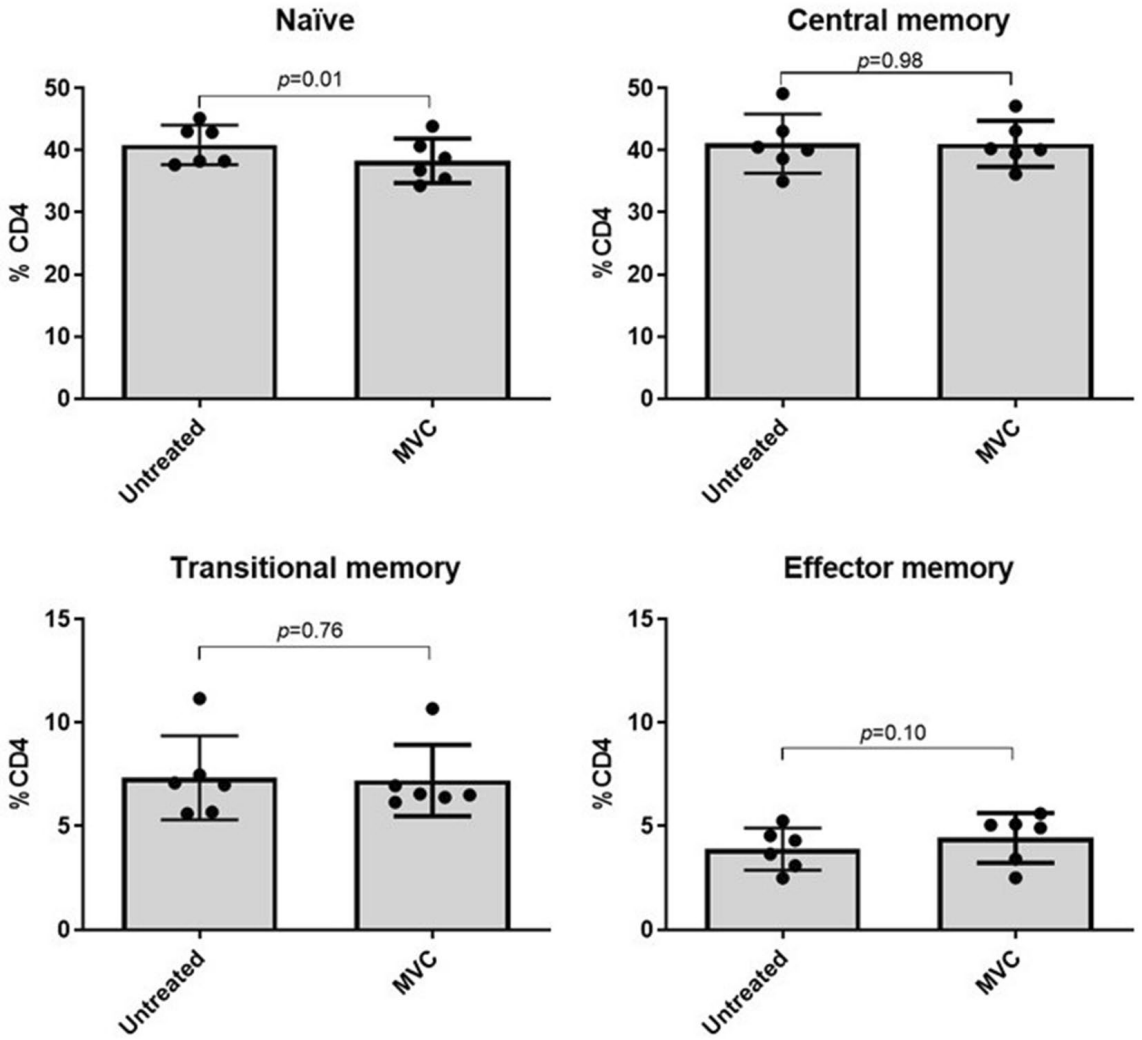
Frequency of CD4 T cells subpopulations. Purified CD4 cells were cultured in absence (untreated) or presence of MVC (5 μM) during 4 days. CD4+ T-cell subpopulations [naïve (CD3+, CD4+, CD45RA+, CCR7+, CD27+), central memory (CD3+, CD4+, CD45RA−, CCR7+, CD27+), transitional memory (CD3+, CD4+, CD45RA−, CCR7−, CD27+) or effector memory (CD3+, CD4+, CD45RA−, CCR7−, CD27−)] were estimated by flow cytometry. Values were summarized as median and interquartile range. The statistical analysis among the conditions was performed with the Wilcoxon test.

We further investigated the impact of MVC on the distribution of CD4 T cell subpopulations. Our findings indicate that MVC did not lead to any noteworthy changes in the frequency of these cell subsets (Fig. 3). Specifically, MVC exhibited no significant effects on the frequency of memory CD4 T cells, including central memory, transitional memory, and effector memory cells. Only a minor reduction in the frequency of naïve cells was observed (*p* = 0.01).

**Figure 4:**
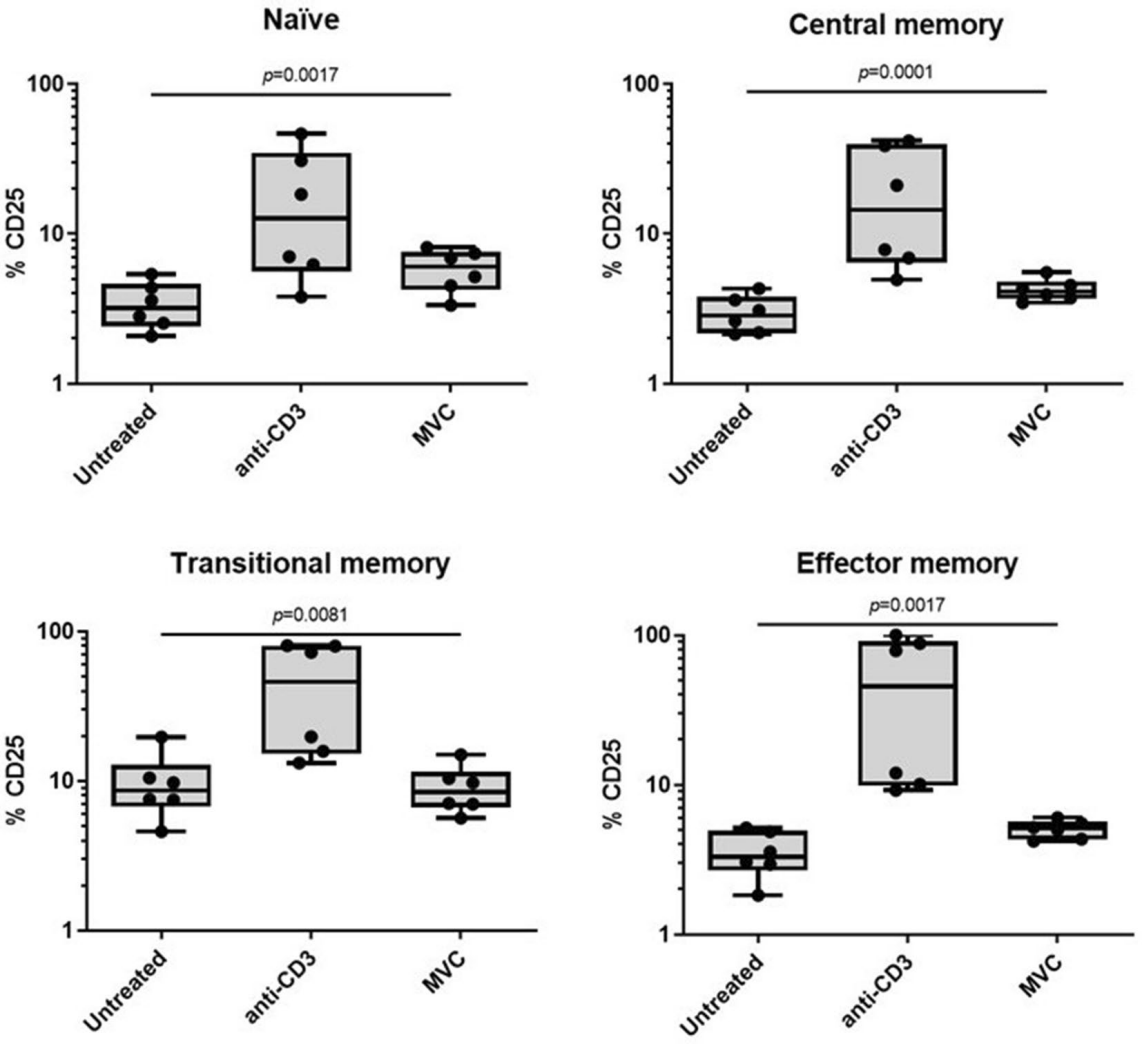
Levels of CD25 activation marker in CD4 T cell subpopulations. Purified CD4 cells were cultured in the absence (untreated) or presence of MVC (5 μM), or anti-CD3 (positive control), during 4 days. CD25 expression in CD4 T cells subpopulations [naïve (CD3+, CD4+, CD45RA+, CCR7+, CD27+), central memory (CD3+, CD4+, CD45RA−, CCR7+, CD27+), transitional memory (CD3+, CD4+, CD45RA−, CCR7−, CD27+) or effector memory (CD3+, CD4+, CD45RA−, CCR7−, CD27−)]. CD25 expression on CD4 T cell subpopulations were determined by flow cytometry. Results are shown as percentage of expression of CD25 in each subpopulation. Values were summarized as median and interquartile range. The statistical analysis among the conditions was performed with the Friedman test. No significant differences between untreated and MVC-treated CD4 T cells were observed.

In keeping with the results on the total pool of CD4+ T cells, we noted that MVC did not modify the CD25 levels compared to untreated cells across all CD4 T cell subpopulations, unlike anti-CD3 antibodies which strongly increased the CD25 levels in all these cell subsets (*p* < 0.05) (Fig. 4).

### MVC maintains the highest anti-HIV activity in memory CD4 T Cells

**Figure 5:**
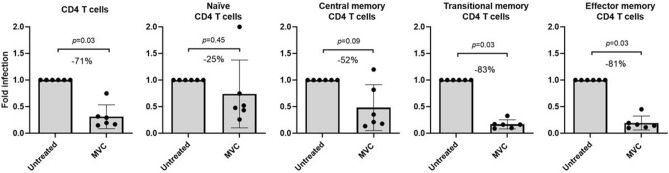
HIV infection levels in CD4 T cells. Purified CD4 cells were cultured in the absence (untreated) or presence of MVC (5 μM) during 4 days. CD4+ T-cell subpopulations were defined as naïve (CD3+, CD4+, CD45RA+, CCR7+, CD27+), central memory (CD3+, CD4+, CD45RA−, CCR7+, CD27+), transitional memory (CD3+, CD4+, CD45RA−, CCR7−, CD27+) or effector memory (CD3+, CD4+, CD45RA−, CCR7−, CD27−). Productive HIV-1 infection was performed in CD4+ T cells with HIV-1 BaL strain (R5 tropic) during 72 h in IL-2-containing medium. Percentage of infected CD4 T cell subsets were estimated by flow cytometry as the percentage of p24-expressing CD4+ T cells. Results are shown as fold-change of infected cells. Values were summarized as median and interquartile range. The statistical analysis among the conditions was performed with the Wilcoxon test.

Additionally, we assessed the antiviral effect of MVC in these experimental conditions that evaluated the immunologic and metabolic parameters on different CD4 T cell subsets. For the infectivity assay, we used a replication competent, multiple round infection, R5-tropic Bal HIV strain. We observed that MVC retains a potent antiviral activity when it was administered before the infection in total CD4 cells (> 70% of inhibition) (*p* < 0.05). Also, we noted a more efficient inhibition of the HIV infection in memory phenotype CD4 T cells, achieving the highest viral inhibition in transitional and effector memory cell subpopulations (> 80%) (Fig. 5).

## Discussion

Previous clinical trials have demonstrated the safety, tolerability, and efficacy of MVC in both ART-naive and ART-experienced people with HIV^38–40^. Due to the well-established tolerability and safety profile, MVC has been proposed as a LRA candidate in the context of the "shock and kill" strategy aimed to cure HIV. In our study, we employed a high MVC concentration (5 μM), which has been demonstrated to induce HIV transcription in absence of cell toxicity in similar experimental conditions^29,33–35^.

Regarding the antiviral activity of MVC, it is noteworthy that the inhibitory concentration required to block HIV entry ranges from 0.5 to 13.4 nM, depending of the specific viral strain utilized in the assays^30^. Our results corroborate the high antiviral activity of MVC, but also indicate that pre-treatment with this drug alone displayed a potent antiviral activity, maintaining a more efficient inhibition of HIV infection on transitional and effector memory cells (Fig. 5). This is consistent with the fact that these CD4 subsets exhibit higher HIV infection levels, as has been previously reported^41^. This is relevant because the HIV reservoir primarily comprises various populations of memory CD4 T cells, including central, transitional, and effector memory cells^4,5^. The dual effect displayed by MVC is of paramount interest because on one hand it is able to reactivate the expression of integrated HIV, acting as an LRA, and on the other hand would block the reinfection of new targets acting as a potent antiviral. In the context of the shock and kill strategy, MVC administration as an LRA would retain its antiviral efficacy that becomes evident when administered before infection, counteracting productive HIV infection in memory CD4 T cells without promoting an increase in infection levels. However, we only have evaluated one R5-tropic HIV strain in our experimental conditions, which could represent a limitation of this work.

Furthermore, our results indicate no differences in activation, differentiation or proliferation of CD4 T cells upon MVC exposure (Figs. 1, 3 and 4). These observations align with previous reports indicating that MVC can be administered in vivo without inducing T cell activation or cellular toxicity. For instance, a gene expression analysis of NF-κB-dependent pro-inflammatory cytokines (gamma interferon, IL-6, IL-10, and tumor necrosis factor-alpha) did not reveal significant differences in the induction of their expression following MVC administration in HIV-infected patients^36^. Another study examined the transcriptomic profile of CD4+ T lymphocytes from MVC-treated patients and found non-significant changes in *TNF* gene expression^42^. Actually, exposure to MVC can decrease the expression of some activation expression markers on T lymphocytes^43^. In the context of LRA, our results are particularly interesting because several HIV latency-reversing agents have shown to increase the levels of activation markers in CD4 T cells. For instance, prostratin, HDAC inhibitors, and bryostatin can elevate CD69 expression in CD4 T cells^44–47^. Additionally, prostratin can increase CD25 and HLA-DR expression levels in naïve and central memory CD4 T cells^47^. Similarly, a clinical trial observed a significant increase in HLA-DR, CD69 or PD-1 expression in CD4+ T cells after romidepsin administration. The largest increase of HLA-DR levels was observed in effector memory and highly differentiated effector CD4 cells^48^.

On the other hand, immunometabolism may play an important role in HIV infection. It has been described that the glucose transporter 1 (GLUT1) expression is necessary for the post-entry steps of HIV-1 replication in CD4+ T cells, and the metabolism of nucleotides is critical for HIV-1 reverse transcription^49,50^. Also, a previous report indicates that increased glycolytic and oxidative rates in CD4 T cells are associated with higher levels of susceptibility to HIV infection^41^. In the context of HIV latency reversing, a prior study reported that treatment with tideglusib (an AKT/mTOR activator capable of inducing HIV reactivation) had a minimal effect on basal CD4 T cell glycolysis^51^. Moreover, a transcriptome analysis has revealed that bryostatin may activate some pathways such as pyrimidine metabolism, purine metabolism and p53 signaling, which would be related to its ability to reactivate HIV latency^52^. To our knowledge, we report for the first time that a drug (approved for clinical use) capable of reactivating HIV latency, such as MVC, does not induce an increase in glycolytic or oxidative metabolic rates (Fig. 2). Our observations are relevant because suggests that MVC would not heighten HIV susceptibility of CD4 uninfected cells or alter the functionality of immune cells involved in HIV infection.

Another challenge in the context of HIV cure is the persistence and maintenance of the HIV reservoir in lymphatic tissues, where antiretroviral drug concentrations are lower compared to the blood, thus failing to achieve complete inhibition of HIV replication^53^. Interestingly, MVC present some additional characteristics that could be beneficial in the context of HIV latency reversion as suggested in previous works from our group and others. For instance, (a) MVC may be administered at higher doses than conventional in HIV infected patients, according to clinical guidelines, and (b) MVC can reach higher concentrations in lymphoid tissues in comparison to other antiretroviral drugs^54^. Therefore, taking these data and our results into consideration, further studies could be performed to evaluate the effect of high doses of MVC on virus replication and the size of the HIV reservoir in lymphoid tissues.

Also, it has been reported that MVC may induce beneficial immunological changes in HIV-1 infection, inducing a good recovery of immune cells such as CD4+ and/or CD8+ lymphocytes from people with HIV^55,56^. In this context, our data suggest that administration of higher concentrations of MVC could also help to counteract residual chronic immune activation/inflammation that persists throughout the HIV disease, which can accelerate non-AIDS-related events and CD4+ T cell depletion^57^. Additionally, considering previous data on the potential benefits of MVC, we believe that MVC administration in CD4 T cells from chronic HIV patients would not alter the levels of activation markers, similar to what was observed in our results in CD4 T cells from HIV seronegative individuals.

In summary, we have observed that a high concentration of MVC, which is able to induce HIV transcription according to previous reports, does not have a detrimental effect on immunological parameters of CD4 T cells and retains its potent antiviral activity against HIV. Also, MVC offers advantages over other HIV latency reversal compounds as it exhibits a dual effect, as an LRA activating HIV expression and as a potent antiviral blocking HIV entry, all without altering activation and metabolic parameters of CD4 T cells. Therefore, our findings provide new evidence to support MVC as a promising LRA for therapeutic interventions aimed at achieving functional HIV cure.

## Methodology

### Cells and culture

Blood samples were obtained from seronegative donors through the French blood bank (Etablissement Français du Sang) in the context of a collaboration agreement with the Institut Pasteur (C CPSL UNT; number 15/EFS/023). All study participants provided informed consent.

Peripheral blood mononuclear cells (PBMCs) were isolated from the blood samples using centrifugation through a Ficoll-Hypaque gradient. Also, the same isolates of PBMCs were used for all the experiments. CD4+ T-cells were purified to a purity level exceeding 90% from freshly isolated PBMCs using negative selection with antibody-coated magnetic beads (EasySep Human CD4+ T-cell Enrichment Kit) within a Robosep instrument (Stem Cell Technology). Purified CD4+ cells were cultured at a concentration of 10^6^ cells/mL in RPMI 1640 medium containing GlutaMAX, 10% fetal calf serum (FCS), penicillin (10 IU/mL), and streptomycin (10 μg/mL). These cells were cultured in the presence or absence of MVC (5 μM) for a duration of 4 days at 37 °C in a humidified atmosphere with 5% CO_2_. MVC 5 μM has previously shown to induce significant viral reactivation in HIV-infected cells^33,34^.

### Evaluation of T cell activation markers

For the analysis of activation markers, CD4+ T-cells were subjected to incubation with the LIVE/DEAD Fixable Aqua Dead Cell Stain Kit (Life Technologies), and subsequently with CD3-eFLuor450 (eBiosciences), CD4-AF700 (eBiosciences), CD69-PE-Cy7 (Becton Dickinson), HLA-DR-FITC (Becton Dickinson), PD-1-PE (eBiosciences), and Ki76-eFluor660 (eBiosciences) antibodies to evaluate the expression of these T-cell activation markers. The Ki67 marker was initially labeled intracellularly. Cells were incubated with the antibodies for a duration of 25 min, followed by thorough washing with phosphate buffered saline (PBS) 1X containing 1% FCS and subsequent fixation in paraformaldehyde (PFA) 4% paraformaldehyde (PFA). The samples were then prepared for flow cytometry analysis utilizing an LSRII device (BD Biosciences).

### Evaluation of CD4 T cell subpopulations

To assess the distribution and evaluate CD25 expression within CD4 T cell subpopulations, CD4+ T-cells underwent incubation with the LIVE/DEAD Fixable Aqua Dead Cell Stain Kit (Life Technologies), as well as a panel of specific antibodies including CD3-eFLuor450 (eBiosciences), CD4-AF700 (eBiosciences), CD45RA-PE-Cy7 (Becton Dickinson), CCR7-PE-ECD (BioLegend), CD27-PE (Becton Dickinson), and CD25-APC (eBiosciences). These antibodies were incubated with the cells and for a duration of 25 min. Subsequently, the cells were thoroughly washed in PBS containing 1% FCS and then fixed in PFA 4% for flow cytometric analysis utilizing an LSRII device (BD Biosciences).

Within the CD4+ T-cell population, distinct subsets, including naïve (Tn; CD3+, CD4+, CD45RA+, CCR7+, CD27+), central memory (Tcm; CD3+, CD4+, CD45RA−, CCR7+, CD27+), transitional memory (Ttm; CD3+, CD4+, CD45RA−, CCR7−, CD27+), and effector memory (Tem; CD3+, CD4+, CD45RA−, CCR7−, CD27−), were estimated through flow cytometry analysis. Additionally, CD25 expression levels were evaluated within each of these subpopulations.

### Evaluation of metabolic fluxes

The oxygen consumption rate (OCR) and extracellular acidification rate (ECAR) were measured utilizing a Seahorse XF96 metabolic analyzer, in strict accordance with the manufacturer's recommended procedure. Briefly, CD4+ T-cells were seeded at a concentration of 3 × 10^5^ cells per well onto XF96 plates (Seahorse Bioscience) that had been pre-coated with 0.5 mg/ml Cell Tack (Corning). Following this, Seahorse XF culture media was promptly added to each well, and the cells were incubated for a duration of 50 min within a CO_2_-free incubator set at a temperature of 37 °C. Subsequently, the plate was loaded into the Seahorse analyzer. The cells were monitored in the Seahorse analyzer upon sequential addition of (1) XFmedia, (2) oligomycin (2.5 μM), (3) FCCP (0.9 μM), and (4) rotenone (1 μM) and antimycin A (1 μM) to facilitate the assessment of metabolic parameters.

### Infectivity assay

The productive HIV-1 infection in vitro was studied in CD4+ T cells (10^6^ cells/mL) with HIV-1 BaL strain (R5 tropic) (10 ng p24/mL). Infections were performed in 96-U-well plates during 72 h in IL-2-containing medium. Cells were labeled with intracellular p24/K57-FITC (Coulter). Then, cells were incubated with LIVE/DEAD Fixable Aqua Dead Cell Stain Kit (Life Technologies) and CD3-eFLuor450 (eBioscience), CD4-alexaFluor700 (BD Biosciences), CD45RA-ECD (Beckman Coulter), CCR7-PE-Cy7 (BioLegend) and CD27-APC (Miltenyi) antibodies to determine infection levels in each CD4+ T-cell subset. Cells were incubated with the antibodies for 25 min and then washed in PBS plus 2% FCS and fixed in PFA 4%. Percentage of infected CD4 T cell subsets (defined in a previous section) were estimated by flow cytometry (BD LSRII, BD bioscience) as the percentage of p24-expressing CD4+ T cells.

### Data analysis and statistics

The data collected through flow cytometry were analyzed using FlowJo software. To draw comparisons among the experimental conditions, statistical analysis was conducted using the Wilcoxon test to compare two conditions, and ANOVA or the Friedman test to compare more than two conditions, depending on the results of the Shapiro–Wilk normality test. Data analysis and visualization were facilitated by GraphPad Prism 8.0 software.

## Data Availability

The data that support the findings of this study are available from the corresponding author, upon reasonable request.

## References

[1] Trickey A (2017). Survival of HIV-positive patients starting antiretroviral therapy between 1996 and 2013: A collaborative analysis of cohort studies. Lancet HIV.

[2] Siliciano JD (2003). Long-term follow-up studies confirm the stability of the latent reservoir for HIV-1 in resting CD4+ T cells. Nat. Med..

[3] Coiras M, López-Huertas MR, Pérez-Olmeda M, Alcamí J (2009). Understanding HIV-1 latency provides clues for the eradication of long-term reservoirs. Nat. Rev. Microbiol..

[4] Chomont N (2009). HIV reservoir size and persistence are driven by T cell survival and homeostatic proliferation. Nat. Med..

[5] Buzón MJ (2014). HIV-1 persistence in CD4+ T cells with stem cell-like properties. Nat. Med..

[6] Henrich TJ (2014). Antiretroviral-free HIV-1 remission and viral rebound after allogeneic stem cell transplantation: Report of 2 cases. Ann. Intern. Med..

[7] Deeks SG (2012). HIV: Shock and kill. Nature.

[8] Richman DD (2009). The challenge of finding a cure for HIV infection. Science.

[9] De la Torre-Tarazona HE (2020). 4-Deoxyphorbol inhibits HIV-1 infection in synergism with antiretroviral drugs and reactivates viral reservoirs through PKC/MEK activation synergizing with vorinostat. Biochem. Pharmacol..

[10] Laird GM (2015). Ex vivo analysis identifies effective HIV-1 latency-reversing drug combinations. J. Clin. Investig..

[11] Darcis G (2017). Reactivation capacity by latency-reversing agents *ex vivo* correlates with the size of the HIV-1 reservoir. AIDS.

[12] Shan L (2012). Stimulation of HIV-1-specific cytolytic T lymphocytes facilitates elimination of latent viral reservoir after virus reactivation. Immunity.

[13] Elliott JH (2014). Activation of HIV transcription with short-course vorinostat in HIV-infected patients on suppressive antiretroviral therapy. PLoS Pathog..

[14] Gutiérrez C (2016). Bryostatin-1 for latent virus reactivation in HIV-infected patients on antiretroviral therapy. AIDS.

[15] Søgaard OS (2015). The depsipeptide romidepsin reverses HIV-1 latency in vivo. PLoS Pathog..

[16] Archin NM (2012). Administration of vorinostat disrupts HIV-1 latency in patients on antiretroviral therapy. Nature.

[17] Archin NM (2014). HIV-1 expression within resting CD4+ T cells after multiple doses of vorinostat. J. Infect. Dis..

[18] Rasmussen TA (2014). Panobinostat, a histone deacetylase inhibitor, for latent virus reactivation in HIV-infected patients on suppressive antiretroviral therapy: A phase 1/2, single group, clinical trial. Lancet HIV.

[19] Gruell H (2022). Effect of 3BNC117 and romidepsin on the HIV-1 reservoir in people taking suppressive antiretroviral therapy (ROADMAP): A randomised, open-label, phase 2A trial. Lancet Microbe.

[20] Elliott JH (2015). Short-term administration of disulfiram for reversal of latent HIV infection: A phase 2 dose-escalation study. Lancet HIV.

[21] Lim SY (2018). TLR7 agonists induce transient viremia and reduce the viral reservoir in SIV-infected rhesus macaques on antiretroviral therapy. Sci. Transl. Med..

[22] Vibholm L (2017). Short-course Toll-like receptor 9 agonist treatment impacts innate immunity and plasma viremia in individuals with human immunodeficiency virus infection. Clin. Infect. Dis..

[23] Grau-Expósito J (2019). Latency reversal agents affect differently the latent reservoir present in distinct CD4+ T subpopulations. PLoS Pathog..

[24] Pardons M, Fromentin R, Pagliuzza A, Routy JP, Chomont N (2019). Latency-reversing agents induce differential responses in distinct memory CD4 T cell subsets in individuals on antiretroviral therapy. Cell Rep..

[25] Van Lint C, Bouchat S, Marcello A (2013). HIV-1 transcription and latency: An update. Retrovirology.

[26] Zhao M (2019). T cell toxicity of HIV latency reversing agents. Pharmacol. Res..

[27] Walker-Sperling VE, Pohlmeyer CW, Tarwater PM, Blankson JN (2016). The effect of latency reversal agents on primary CD8+ T cells: Implications for shock and kill strategies for human immunodeficiency virus eradication. EBioMedicine.

[28] Woollard SM, Kanmogne GD (2015). Maraviroc: A review of its use in HIV infection and beyond. Drug Des. Dev. Ther..

[29] Walker DK (2005). Species differences in the disposition of the CCR5 antagonist, UK-427,857, a new potential treatment for HIV. Drug Metab. Dispos..

[30] Dorr P (2005). Maraviroc (UK-427,857), a potent, orally bioavailable, and selective small-molecule inhibitor of chemokine receptor CCR5 with broad-spectrum anti-human immunodeficiency virus type 1 activity. Antimicrob. Agents Chemother..

[31] García-Perez J (2011). New insights into the mechanisms whereby low molecular weight CCR5 ligands inhibit HIV-1 infection. J. Biol. Chem..

[32] García-Perez J (2015). A single-residue change in the HIV-1 V3 loop associated with maraviroc resistance impairs CCR5 binding affinity while increasing replicative capacity. Retrovirology.

[33] López-Huertas MR (2017). The CCR5-antagonist Maraviroc reverses HIV-1 latency in vitro alone or in combination with the PKC-agonist Bryostatin-1. Sci. Rep..

[34] López-Huertas MR (2020). Maraviroc reactivates HIV with potency similar to that of other latency reversing drugs without inducing toxicity in CD8 T cells. Biochem. Pharmacol..

[35] Vicenti I (2021). Maraviroc as a potential HIV-1 latency-reversing agent in cell line models and ex vivo CD4 T cells. J. Gen. Virol..

[36] Madrid-Elena N (2018). Maraviroc is associated with latent HIV-1 reactivation through NF-κB activation in resting CD4+ T cells from HIV-infected individuals on suppressive antiretroviral therapy. J. Virol..

[37] López-Huertas MR (2020). Prolonged administration of maraviroc reactivates latent HIV in vivo but it does not prevent antiretroviral-free viral rebound. Sci. Rep..

[38] Gulick RM (2008). Maraviroc for previously treated patients with R5 HIV-1 infection. N. Engl. J. Med..

[39] Cooper DA (2010). Maraviroc versus efavirenz, both in combination with zidovudine-lamivudine, for the treatment of antiretroviral-naive subjects with CCR5-tropic HIV-1 infection. J. Infect. Dis..

[40] Stellbrink HJ (2016). Once-daily maraviroc versus tenofovir/emtricitabine each combined with darunavir/ritonavir for initial HIV-1 treatment. AIDS.

[41] Valle-Casuso JC (2019). Cellular metabolism is a major determinant of HIV-1 reservoir seeding in CD4+ T cells and offers an opportunity to tackle infection. Cell Metab..

[42] Winters MA, Van Rompay KKA, Kashuba ADM, Shulman NS, Holodniy M (2010). Maternal-fetal pharmacokinetics and dynamics of a single intrapartum dose of maraviroc in rhesus macaques. Antimicrob. Agents Chemother..

[43] Arberas H (2013). In vitro effects of the CCR5 inhibitor maraviroc on human T cell function. J. Antimicrob. Chemother..

[44] Rasmussen TA (2013). Comparison of HDAC inhibitors in clinical development: Effect on HIV production in latently infected cells and T-cell activation. Hum. Vaccin Immunother..

[45] Albert BJ (2017). Combinations of isoform-targeted histone deacetylase inhibitors and bryostatin analogues display remarkable potency to activate latent HIV without global T-cell activation. Sci. Rep..

[46] Matsuda K (2021). A therapeutic strategy to combat HIV-1 latently infected cells with a combination of latency-reversing agents containing DAG-lactone PKC activators. Front. Microbiol..

[47] Zerbato JM, McMahon DK, Sobolewski MD, Mellors JW, Sluis-Cremer N (2019). Naive CD4+ T cells harbor a large inducible reservoir of latent, replication-competent human immunodeficiency virus type 1. Clin. Infect. Dis..

[48] Rosás-Umbert M (2020). In vivo effects of romidepsin on T-cell activation, apoptosis and function in the BCN02 HIV-1 Kick&Kill clinical trial. Front. Immunol..

[49] Loisel-Meyer S (2012). Glut1-mediated glucose transport regulates HIV infection. PNAS.

[50] Amie SM, Noble E, Kim B (2013). Intracellular nucleotide levels and the control of retroviral infections. Virology.

[51] Gramatica A (2021). Evaluating a new class of AKT/mTOR activators for HIV latency reversing activity ex vivo and in vivo. J. Virol..

[52] Li BX (2020). Novel pathways of HIV latency reactivation revealed by integrated analysis of transcriptome and target profile of bryostatin. Sci. Rep..

[53] Fletcher CV (2014). Persistent HIV-1 replication is associated with lower antiretroviral drug concentrations in lymphatic tissues. Proc. Natl. Acad. Sci. USA.

[54] Fletcher CV (2022). Persistent HIV transcription and variable antiretroviral drug penetration in lymph nodes during plasma viral suppression. AIDS.

[55] Funderburg N (2010). Effects of maraviroc and efavirenz on markers of immune activation and inflammation and associations with CD4+ cell rises in HIV-infected patients. PLoS ONE.

[56] Pulido I (2012). T-cell changes after a short-term exposure to maraviroc in HIV-infected patients are related to antiviral activity. J. Infect..

[57] Lv T, Cao W, Li T (2021). HIV-related immune activation and inflammation: Current understanding and strategies. J. Immunol. Res..

