# Author Correction: High concentrations of Maraviroc do not alter immunological and metabolic parameters of CD4 T cells

**DOI:** 10.1038/s41598-024-67334-w

**Published:** 2024-07-17

**Authors:** Erick De La Torre Tarazona, Caroline Passaes, Santiago Moreno, Asier Sáez-Cirión, José Alcamí

**Affiliations:** 1 Infectious Diseases Department, Instituto Ramón y Cajal de Investigación Sanitaria (IRYCIS), University Hospital Ramón y Cajal, Madrid, Spain; 2 HIV, Inflammation and Persistence Unit, Institut Pasteur, Université Paris Cité, Paris, France; 3 Viral Reservoirs and Immune Control Unit, Institut Pasteur, Université Paris Cité, Paris, France; 4 AIDS Immunopathogenesis Unit, National Center of Microbiology, Instituto de Salud Carlos III (ISCIII), Madrid, Spain; 5 Centro de Investigación Biomédica en Red de Enfermedades Infecciosas (CIBERINFEC), Instituto de Salud Carlos III (ISCIII), Madrid, Spain; 6 Department of Medicine, Alcalá University, Madrid, Spain

Correction to: *Scientific Reports* 10.1038/s41598-024-64902-y, published on 17 June 2024

The original version of this Article contained an error in the Acknowledgements section.

“EDLTT was supported by the Sara Borrell program (CD21/00102, Ministry of Science and Innovation from the Spanish Government). We also thank to GILEAD Sciences Fellowship (GLD 18/0090).”

now reads:

“EDLTT was supported by the Sara Borrell program (CD21/00102, Ministry of Science and Innovation from the Spanish Government). We also thank to GILEAD Sciences Fellowship (GLD 18/0090). This study has been funded by Instituto de Salud Carlos III (ISCIII) through the project PI20/00945 and co-funded by the European Union.”

The original Article has been corrected.

